# Peripheral Blood-Derived Stem Cells for the Treatment of Cartilage Injuries: A Systematic Review

**DOI:** 10.3389/fbioe.2022.956614

**Published:** 2022-07-22

**Authors:** Yanlin Zhu, Weili Fu

**Affiliations:** Department of Orthopedics, Orthopedic Research Institute, West China Hospital, Sichuan University, Chengdu, China

**Keywords:** peripheral blood-derived stem cells, cartilage injuries, PBMSC, BMSC, PBMNCs

## Abstract

**Background:** The treatment of cartilage damage is a hot topic at present, and cell therapy is an emerging alternative therapy. Stem cells derived from peripheral blood have become the focus of current research due to the ease of obtaining materials and a wide range of sources.

**Methods:** We used a text search strategy using the [“mesenchymal stem cells” (MeSH term) OR “MSC” OR “BMMSC” OR “PBMSC” OR” PBMNC” OR “peripheral blood stem cells”] AND (cartilage injury [MeSH term] OR “cartilage” OR “chondral lesion”). After searching the literature, through the inclusion and exclusion criteria, the last included articles were systematically reviewed.

**Result:** We found that peripheral blood-derived stem cells have chondrogenic differentiation ability and can induce chondrogenic differentiation and repair *in vivo* and have statistical significance in clinical and imaging prognosis. It is an improvement of academic differences. Compared with the bone marrow, peripheral blood is easier to obtain, widely sourced, and simple to obtain. In the future, peripheral blood will be a more potential cell source for cell therapy in the treatment of cartilage damage.

**Conclusion:** Stem cells derived from peripheral blood can repair cartilage and are an important resource for the treatment of cartilage damage in the future. The specific mechanism and way of repairing cartilage need further study.

## 1 Introduction

Cartilage is a special, low-friction articular surface tissue that is essential for weight absorption and smooth gliding of the articulating surfaces in diarthrodial joints, whose primary function is to absorb, cushion, and protect the underlying bone from the forces that arise when the joint is being used. Chondral lesions can lead to direct contact with bone, ultimately leading to osteoarthritis (Rackwitz et al., 2014). Due to the lack of native blood vessels and lymphatic return, the spontaneous healing capacity of cartilage is low and is generally replaced by fibrocartilage (Frisch et al., 2017a). The newly generated fibrocartilage can withstand far less mechanical stress than the original cartilage tissue (Hunziker, 2002). Numerous studies have reported that the newly formed fibrocartilage tends to deteriorate over time (Orth et al., 2014). Therefore, the treatment of chondral lesions is currently an important research topic in traumatology.

Conservative treatment of cartilage damage usually includes corticosteroids, nonsteroidal anti-inflammatory drugs, hyaluronan, and polysulfated glycosaminoglycan (Ferris et al., 2011). However, the abovementioned drugs can only control the symptoms and cannot prevent the occurrence of osteoarthritis (Frisbie et al., 2009). Marrow stimulation techniques, including microfracture and microdrilling, have been widely reported as promoting chondral healing, with microfracture being the most commonly performed (Madry et al., 2011). It penetrates the underlying subchondral bone marrow through drilling, allowing bone marrow mesenchymal stem cells (MSC) and other progenitor cells to enter the cartilage defect for repair and present good clinical outcomes (Bieback et al., 2008). However, after bone marrow stimulation, the joint normally covered by hyaline cartilage is repaired by fibrocartilage, which is biochemically and mechanically inferior to hyaline cartilage (Saris et al., 2009; Seol et al., 2012; Jiang and Tuan, 2015). Continued stress can lead to tissue degeneration and deteriorating results in the long term (Vinatier et al., 2009). Therefore, improving the quality of prosthetic tissue has become a new issue.

The application of autologous mesenchymal stem cells in the joint cavity shows the effect of enhancing cartilage repair in a lasting way (Saw et al., 2013; Skowroński and Rutka, 2013; Reissis et al., 2016). Thus, lately researchers have focused on cell therapy as a therapeutic alternative (Brittberg et al., 1994). There are many sources of mesenchymal stem cells, including bone marrow, adipose tissue, skin, or peripheral blood, or from an umbilical cord donor (Kassis et al., 2006; Larochelle et al., 2006; Huang et al., 2009). While bone marrow (BM) MSCs show a decline in the number and differentiation potential of MSCs with aging or transformation in long-term *in vitro* culture, the peripheral blood mononuclear cell fraction has been shown to enhance cartilage repair in an ovine osteochondral defect model (Emadedin et al., 2012; Hopper et al., 2015a). The use of peripheral blood may provide workable and less invasive translational procedures as this resource also contains MSC with the same potency for chondrogenic differentiation as that of bone marrow MSC (Zvaifler et al., 2000; Huang et al., 2009; Raghunath et al., 2010; Al Faqeh et al., 2012). The purpose of this systematic review is to evaluate the potential of peripheral blood-derived stem cells in the treatment of cartilage injury by collecting relevant literature on the treatment of cartilage injury with peripheral blood-derived stem cells in the past two decades, including *in vitro* and *in vivo* experimental articles.

## 2 Materials and Methods

### 2.1 Data Sources and Search Strategy

We conducted a systematic review based on the PRISMA (Preferred Reporting Items for Systematic Review and Meta-Analysis) guidelines (Moher et al., 2009). We used a text search strategy using the [“mesenchymal stem cells” (MeSH term) OR “MSC” OR “BMMSC” OR “PBMSC” OR” PBMNC” OR “peripheral blood stem cells”] AND [cartilage injury (MeSH term) OR “cartilage” OR “chondral lesion”]. Specifically, we searched the PubMed, Embase, and OVID databases from inception to 20 April 2022. We also assessed the bibliographies of identified studies to seek additional articles. We did not add language restrictions.

Along with the database search, we examined the references of included studies and previously published systematic reviews to identify additional studies. We also checked the International Clinical Trials Registry Platform Search Portal and ClinicalTrials.gov (https://clinicaltrials.gov/) to identify the currently ongoing or recently completed trials.

### 2.2 Inclusion and Exclusion Criteria

#### 2.2.1 Inclusion

1. Any basic English-language scientific studies of the PB-derived primitive cells that exhibited chondrogenic or multipotent mesenchymal differentiation abilities.
2. *in vivo* animals using PB as a source of chondrogenic progenitor cells for cartilage regeneration were also included.
3. Human studies using PB as a source of chondrogenic progenitor cells for cartilage regeneration were also included.
4. Any study that has at least one outcome that can be documented.

### 2.3 Exclusion

Any studies of primitive cells that were not chondrogenic or not derived from the PB and *in vivo* studies that only used non-PB sources were excluded.

### 2.4 Quality Assessment

The risk of bias graph in Review Manager 5.3 was used to evaluate the methodologic quality of included RCT studies in this systematic review. This seven-element checklist qualitatively assesses various aspects of trial quality (random sequence generation, allocation concealment, blinding of participant and personnel, blinding of outcome assessment, incomplete outcome data, selective reporting, and other bias) using an ordinal scoring system comprising high risk, low risk, or unclear risk response options for each statement in Review Manager 5.3. A higher score obtained with the Review Manager 5.3 is indicative of higher methodological study quality. We did not assess publication bias with a funnel chart because we had less than 10studies for each comparison in this review.

QUADAS (Quality Assessment of Diagnostic Accuracy Studies) was used to evaluate the methodologic quality of other studies. The detailed items of the scale are as follows:

1. Was a consecutive or random sample of patients enrolled? Yes/No/Unclear
2. Was a case-control design avoided? Yes/No/Unclear
3. Did the study avoid inappropriate exclusions? Yes/No/Unclear
4. Could the selection of patients have introduced bias? RISK: LOW/HIGH/UNCLEAR
5. Is there a concern that the included patients do not match the review question? CONCERN: LOW/HIGH/UNCLEAR
6. Were the index test results interpreted without the knowledge of the results of the reference standard? Yes/No/Unclear
7. If a threshold was used, was it prespecified? Yes/No/Unclear
8. Could the conduct or interpretation of the index test have introduced bias? RISK: LOW /HIGH/UNCLEAR
9. Is there a concern that the index test, its conduct, or interpretation differ from the review question? CONCERN: LOW /HIGH/UNCLEAR
10. Is the reference standard likely to correctly classify the target condition? Yes/No/Unclear
11. Were the reference standard results interpreted without the knowledge of the results of the index test? Yes/No/Unclear
12. Could the reference standard, its conduct, or its interpretation have introduced bias? RISK: LOW /HIGH/UNCLEAR
13. Is there a concern that the target condition, as defined by the reference standard, does match the review questions? CONCERN: LOW /HIGH/UNCLEAR
14. Was there an appropriate interval between index test(s) and reference standard? Yes/No/Unclear
15. Did all patients receive reference standard? Yes/No/Unclear
16. Did patients receive the same reference standard? Yes/No/Unclear
17. Were all patients included in the analysis? Yes/No/Unclear
18. Could the patient flow have introduced bias? RISK: LOW /HIGH/UNCLEAR

### 2.5 Data Extraction

A single reviewer screened all the citations and abstracts generated by the literature search and applied the selection criteria. Identified randomized trials were assessed for inclusion by two reviewers. Any disagreement between them on the eligibility of certain studies was resolved through discussion with a third reviewer. The titles of journals and names of authors were not masked during the study selection process.

Each investigator independently extracted the following data:

1. Study characteristics, including species, number, character of included species, type of study, evaluation method, injury site, and degree of damage.
2. Experimental details including cell source, cultivation and extraction methods, cell character markers, number of cells, cell implantation method, and surgical approach.
3. Experimental results and adverse events.

## 3 Result

### 3.1 Basic Characteristic

**FIGURE 1:**
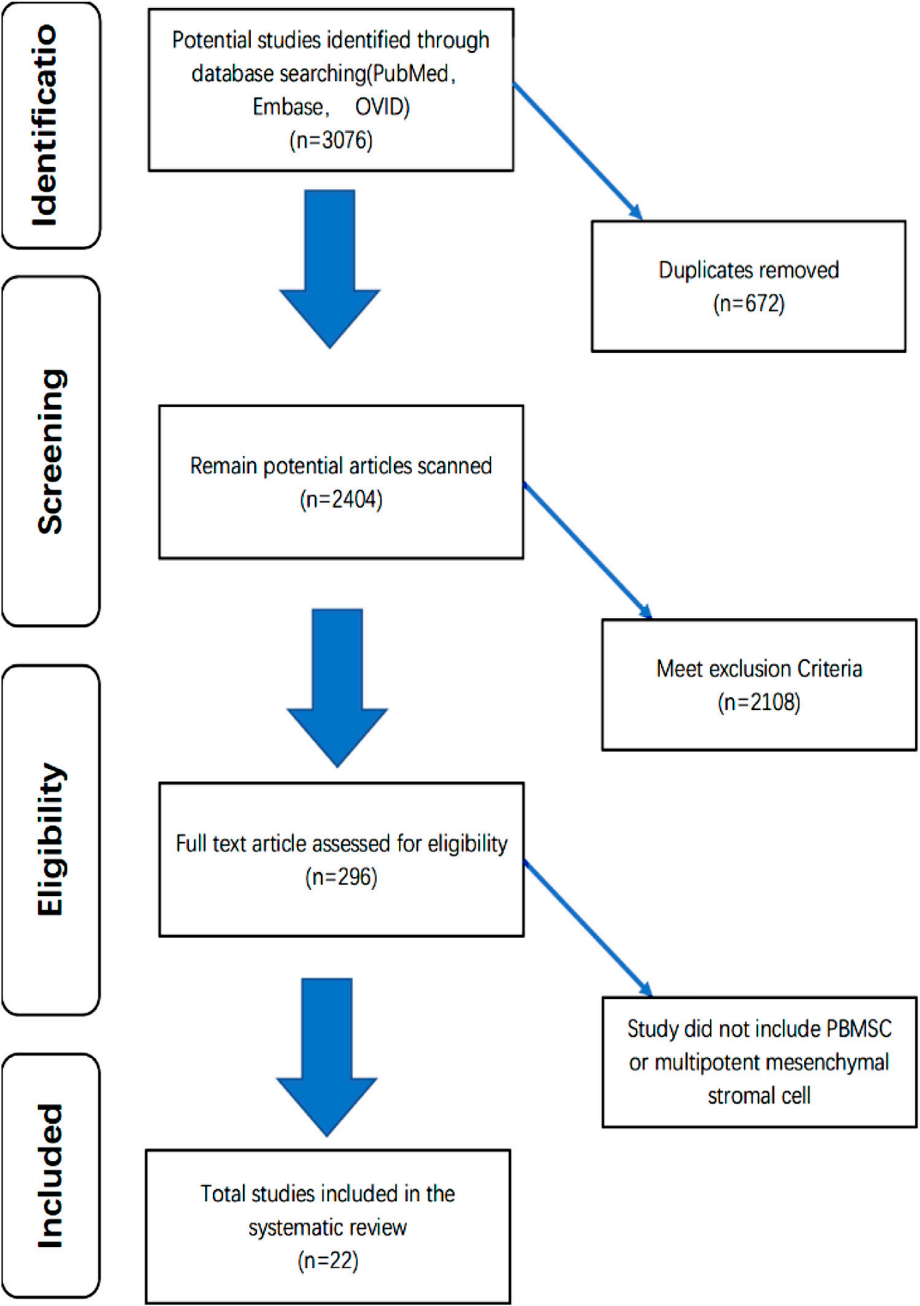
Flow Chart.

**FIGURE 2:**
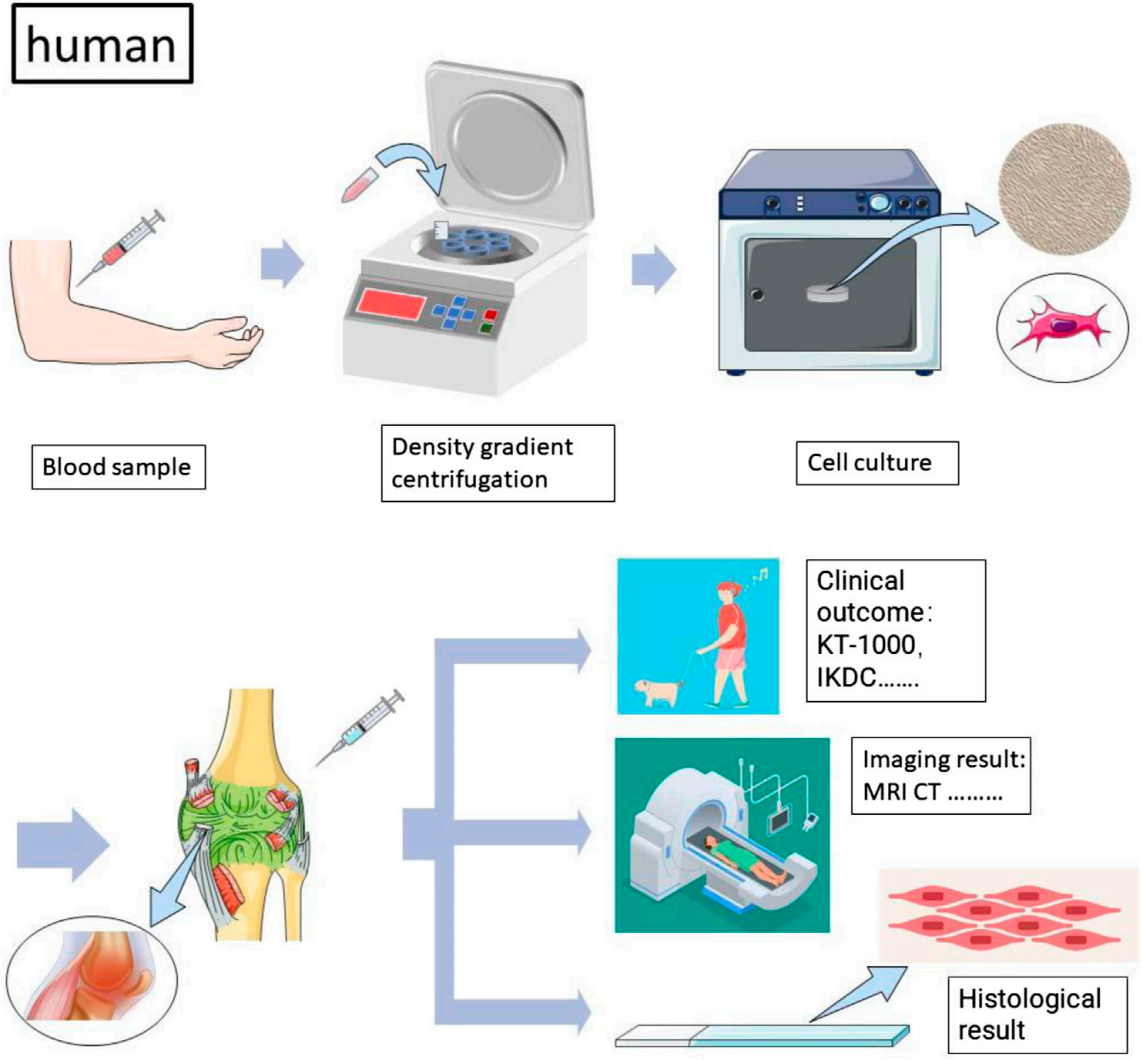
The general process of human peripheral blood-derived stem cell experiments (in vivo).

**FIGURE 3:**
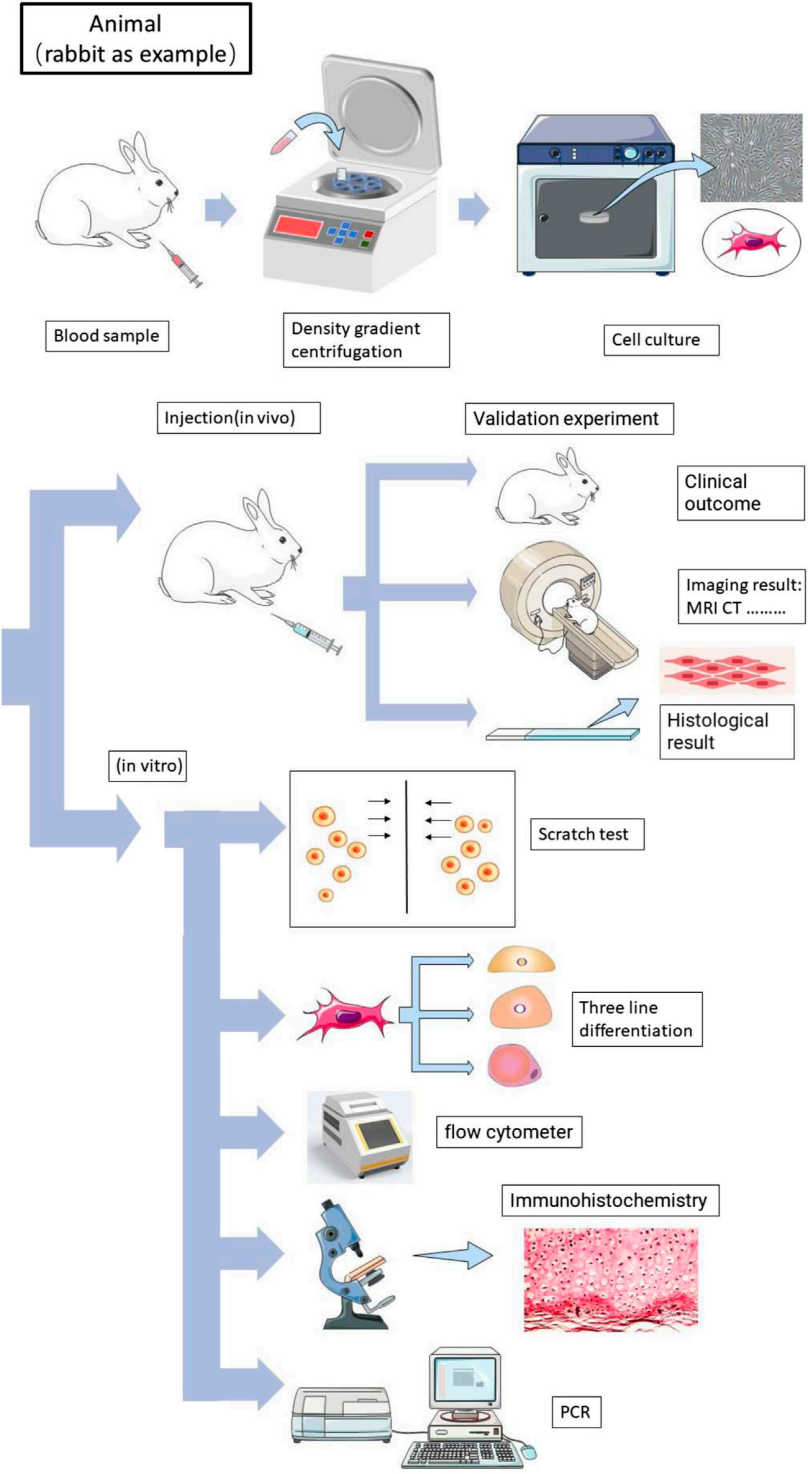
The general process of animal and *in vitro* peripheral blood-derived stem cell experiments (rabbit as an example).

**FIGURE 4:**
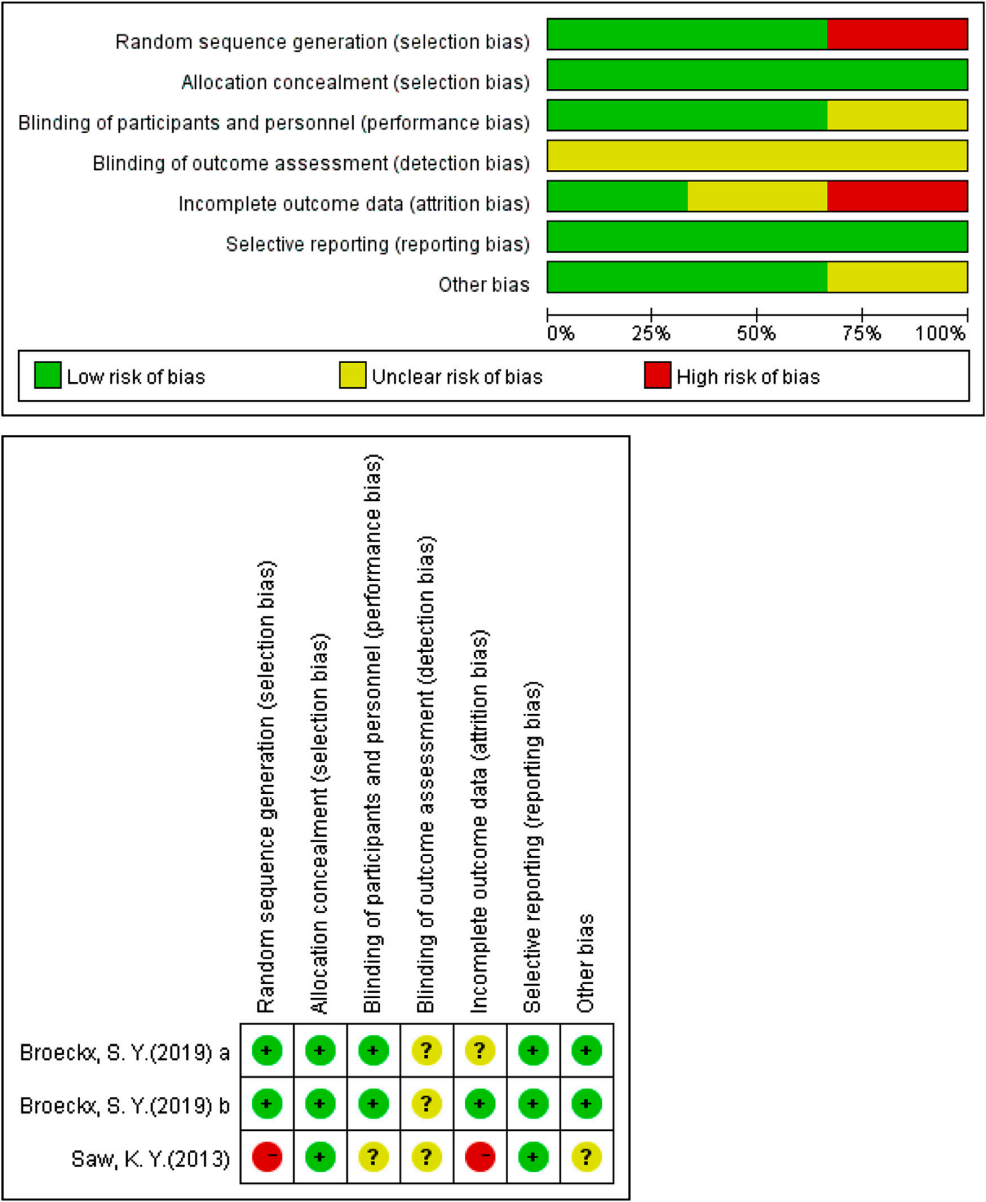
Risk of bias with RCTs.

According to our retrieval strategy abovementioned, we retrieved a total of 3,076 articles. After a brief reading of the abstracts and titles, duplicate articles and irrelevant articles were excluded, and a total of 296 articles were reviewed in detail (Figure 1). After excluding articles that do not contain related stem cells, we ultimately included 24 articles between 2008 and 2022 for the systematic review (Jancewicz et al., 1995; Pufe et al., 2008; Saw et al., 2011; Casado et al., 2012; Chong et al., 2012; Kim et al., 2012; Skowroński et al., 2012; Saw et al., 2013; Skowroński and Rutka, 2013; Turajane et al., 2013; Broeckx et al., 2014a; Fu et al., 2014a; Broeckx et al., 2014b; Fu et al., 2014b; Turajane et al., 2014; Hopper et al., 2015b; Frisch et al., 2017b; Broeckx et al., 2019a; Broeckx et al., 2019b; Monckeberg et al., 2019; Ying et al., 2020; Henson et al., 2021). The data from 24 studies were analyzed, including seven fully *in vitro* studies and 17 *in vivo* studies (Table 1). The experimental subject includes humans, sheep, rabbits, and horses. A total of nine articles included *in vitro* experiments, all (100%) of which confirmed the tendency of peripheral blood-derived stem cells to differentiate into chondrocytes. In terms of cell sources, 10 articles used G-CSF-stimulated PBMSCs, four articles used chondro-induced PBMSCs, and eight articles directly used the peripheral blood stem cells after apheresis or gradient centrifugation. *In vivo* experiments include three comparative studies, one prospective study, three RCTs, five case reports, one preliminary study, and one pilot study. Cartilage defects in nine of the studies were graded with ICRS and were all greater than grade 3. All characteristics of the included literature are listed in Tables 1, 2, 3. Figures 2, 3 demonstrates the basic experimental procedure. Table 1 and Figure 4 show the methodological quality evaluation results. The detailed results of the quality evaluation are shown in Figure 4 and Table 4.

**TABLE 1:** PBSCs in animals.

First author	Species	Number	Character	Type of study	Evaluation method	Injury site(number)	Degree of damage
Henson et al. (2021)	Welsh Mountain female sheep	40	3–4 year-old (adult) (mean age 3.2 years), 40–42 kg	Comparative study	MRI, Gross Morphology, Histology, and Immunohistochemistry	the medial femoral condyle	Full-thickness chondral defects of 8 mm diameter
Broeckx et al. (2019)	Horse	75	22 mares, 16 geldings and 37 stallions	RCTs	visual lameness assessment, flexion test	Fetlock joint	Early staged fetlock degenerative joint disease
Broeckx et al. (2019)	horse	12	3 geldings and 9 mares (median age 8.5 years)	RCTs	weekly joint assessment, AAEP score, an inertial sensor-based system, X-ray, Synovial fluid analysis, OARSI, and Immunohistochemistry	Metacarpophalangeal OA	surgically induced OA
Fu et al. (2014)	Rabbit	30	New Zealand White rabbits, aged about 4 months	Controlled laboratory study	histological scoring, histochemical staining, and immunohistochemistry	the trochlear groove of the distal femur	Full-thickness articular osteochondral defects (5 mm in diameter and 1–2 mm in depth)
Broeckx et al. (2014)	Horse	50	clinical lameness for at least 3 months	Preliminary study	Cytological Staining, Immunocytochemistry, Flow Cytometry, RT-PCR, and AAEP	fetlock	NA
Broeckx et al. (2014)	Horse	165	NA	Pilot study	Clinical lameness; locomotory disorder; and positive flexion test	Stifle joint (30), fetlock joint (58), coffin joint (43), pastern joint (34)	Degenerative joint disease

First author	Cell source	Cultivation and extraction methods	Cell character markers	number of cells	Cell implantation method	Surgical approach
Henson et al. (2021)	Autologous G-CSF activated PB	Apheresis	*CD34*, *CD45*, *CD73*, *CD90, and CD105*	NA	intra-articular injections	intra-articular injections
Broeckx et al. (2019)	Chondrogenic induced PBMSCs	DGC and PA	*CD45*, *MHC II*, *CD29*, *CD44, and CD90*	2 × 10^6^ cells/ml	Articular injection	Articular injection
Broeckx et al. (2019)	Chondrogenic induced PBMSCs	DGC and PA	Aggrecan+, Col II+, COMP+, p63+ and GAG+; decrease in Ki67.	2 × 10^6^ cells in/ml	Articular injection	Articular injection
Fu et al. (2014)	Autologous G-CSF activated PB	Erythrocyte Lysis and PA	*CD44*, *CD45*, and *MHC II*	4 × 10^6^ cells/ scaffold	Surgical implantation	Establishment of animal model
Broeckx et al. (2014)	PB-MSCs (native or chondrogenic induction)	DGC and PA	Col II, Ki67 p63, vimentin, and MHCII aggrecan	NA	Single IA injection	PB-MSCs with or without PRP injection
Broeckx et al. (2014)	PB-MSCs (native or chondrogenic induced)	DGC and PA	Col II, Ki67 p63, vimentin, and MHCII aggrecan	NA	Single IA injection	PB-MSCs with PRP injections

First author	Time	Postoperative treatment	Clinical outcome	Imaging results	Experimental results	Adverse event
Henson et al. (2021)	8 weeks	NA	NA	SPION labeled cells could not be detected within the defect at any of the time points studied despite using MRI sequences	ICRS found No significant difference between treatment groups the repaired tissue was fibrocartilagenous in nature rather than hyaline cartilage	no adverse event
Broeckx et al. (2019)	1 year	Dexmedetomidine hydrochloride and Ketoprofen	Improved AAEP score*, Flexion score* and Pain score*	NA	NA	3 mild infections of the upper respiratory tract
Broeckx et al. (2019)	11 weeks	Dexmedetomidine hydrochloride	AAEP scores in week 7*	radiographic changes were not significantly different	higher viscosity score*, less wear lines were present in the intervention group*, higher COMP in the cartilage adjacent*	1 horse had an increase in local temperature 2 horses had a limited range of motion
Fu et al. (2014)	24 weeks	allowed to move freely and had free access to food pellets and water	NA	NA	PB MSCs had a greater chondrogenic ability than BM MSCs* The histological scores were significantly better in PB MSCs* defects were synthesized with abundant cartilage matrices with a regular arrangement in PB MSCs	NA
Broeckx et al. (2014)	12 months	NA	Improved short- and long-term clinical evolution scores*; Relief from clinical lameness, flexion pain and joint effusion	NA	exhibiting increases in the levels of Col II, aggrecan and cartilage oligomeric matrix protein	NA
Broeckx et al. (2014)	18 weeks	NA	Improved short- and long-term clinical evolution scores*; Relief from clinical lameness and locomotor disorder	NA	NA	Moderate flare reaction (without long-term effects, 3 horses)

DGC, density gradient centrifugation; PA, plastic adherence; AAEP, American association of equine practitioners; OARSI, the Osteoarthritis research society international; OA, osteoarthritis; COMP, cartilage oligomeric matrix protein; NI, not involving; AAV, human adeno-associated virus; rAAV:recombinant AAV; AAPBSC, autologous activated peripheral blood stem cells; IA, intraarticular; rt-PCR, reverse transcriptase-polymerase chain reaction; HHS, the Harris Hip score. * means statistically different.

**TABLE 2:** PBSCs in human.

First author	Species	Number	Character	Type of Study	Evaluation method	Injury site(number)	Degree of damage
Ying et al. (2020)	Human	37(15 males)	age range 31–64 years	prospective study	HHS, µCT Scanning, Histochemistry, Immunohistochemistry (IHC), and Immunofluorescence analyses,	hip	microfracture and/or cystic degeneration existed between cartilage and subchondral bone
Monckeberg et al. (2019)	Human	20	7 women and 13 man with average age of 32.7	Comparative study	IKDC, VAS, MRI, and ICRS	1.Trochlea(9) 2.Femoral condyle(5) 3.Patella(6)	ICRS grade>3
Fu et al. (2014)	Human	1	19 years old	case report	X-rays, CT and MRI, Tegner, Lysholm, and IKDC 2000 scores.	Lateral femoral trochlea	Full-thickness cartilage defects(ICRS grade IV)
Turajane et al. (2013)	Human	5	52–59 years old	Case report	WOMAC and KOOS	Medial condyle (4) and patellofemoral (1)	Early-stage OA(ICRS grade III and IV)
Skowroński and Rutka. (2013)	Human	46	7–52 years old (average age:26 years)	Comparative study	KOOS and Lysholm and VAS scales	Medial femoral condyle	Osteochondral lesions(ICRS grade IV)
Saw et al. (2013)	Human	50	22–50 years old	RCTs	IKDC, MRI scan, and ICRS	Knee	Chondral defects(ICRS grade III and IV)
Skowroński et al. (2012)	Human	52	16–55 years old	Case report	KOOS, Lysholm and VAS scales, and MRI	Patella (22), medial femoral condyle (38), and lateral femoral condyle (6)	Cartilage lesions (ICRS grade III and IV)
Saw et al. (2013)	Human	5	19–52 years old	case report	Second-Look Arthroscopy and Histology	Knee	Chondral defects (ICRS grade III and IV)
Jancewicz, P.(2004)	Human	9	NA	Case report	clinical examination, Magee score, and MRI	Talus	Osteochondral defects(ICRS IV)

First author	Cell source	Cultivation and extraction methods	Cell character markers	Number of cells	Cell implantation method	Surgical approach
Ying et al. (2020)	G-CSF activated PB	apheresis	NA	minimum concentration of 8 × l0^6^/l	arterial injection	infused through the medial circumflex femoral artery.
Monckeberg et al. (2019)	PBSCs	autologous PB (Apheresis)	N/A	430,000 PBSC ± 270.000/ml	articular injection with PRP	knee arthroscopy
Fu et al. (2014)	Autologous G-CSF mobilized PB	Blood cell separation	NA	3.496 × 10^7^ cells/ml	Surgical implantation	Debridement + PBSCs with autologous periosteum flap cover
Turajane et al. (2013)	Autologous G-CSF activated PB	Leukapheresis	*CD34* ^*+*^ *CD105* ^*+*^	2.67–5.99 × 10^3^ cells/injection	Repeated IA injections	Debridement + BMS + repeated IA injections
Skowroński and Rutka. (2013)	Autologous G-CSF activated PB,	NA	NA	1.25 × 10^6^–5.2 × 10^6^ cells/ml	Surgical implantation	Debridement + modified sandwich technique
Saw et al. (2013)	Autologous G-CSF mobilized PB	Apheresis	NA	NA	IA injections	IA injections
Skowroński et al. (2012)	Autologous G-CSF mobilized PB	Apheresis	NA	2.0 × 10^7^ cells/injection	Repeated IA injections	Debridement + BMS + HTO(1) + repeated IA injections
Saw et al. (2013)	Autologous G-CSF activated PB	Blood cell separation	*CD34* ^*+*^	NAC	Surgical implantation	Debridement + sandwich technique

First author	Time	Postoperative treatment	Clinical outcome	Imaging results	Experimental results	Adverse event
Ying et al. (2020)	36 months	NA	no significant difference in HHSs between the two groups at 36 months	no significant differences between the control group and the combination treatment group in µCT Scanning,	no significant difference in osteoclast number was found between the control group and the combination treatment group	
Monckeberg et al. (2019)	5 years	Nonsteroidal anti-inflammatory drugs and acetaminophen, rehabilitation protocol	Improved IKDC score* and lower VAS score*	improved	Improved ICRS score*	2: myalgia and fever
Fu et al. (2014)	7.5 years	Strict rehabilitation program	improved IKDC 2000 subjective score*, Lysholm score and Tegner score*	CT: subchondral bone Recovery; MRI: near-normal cartilagelike tissue regeneration	Regenerated articular cartilage with a smooth surface, but with a slightly yellowish and shallow morphology	NA
Turajane et al. (2013)	6 months	Nonweight bearing	Improved WOMAC and KOOS* Succeeded in regenerating articular cartilage	NA	NA	Mild swelling and discomfort
Skowroński and Rutka. (2013)	5 years	Passive and active exercises, nonweight to full-weight bearing	Improved KOOS and Lysholm scales, relief of VAS scale*;	92% of patients with good results MRI: satisfactory reconstruction of the cartilaginous surface and good regenerative integration	NA	NA
Saw et al. (2013)	18 months	NA	No IKDC score difference compared to the control group	Improved MRI morphologic scores	Improved total ICRS II histologic scores	Deep vein thrombosis (1 patient in the control group)
Saw et al. (2013)	10–26 months	crutch assisted partial to full weight-bearing	NA	X-ray: reappearance of medial articulation	generated full-thickness articular hyaline cartilage	Minimal discomfort from PBSCs harvesting and IA injection

* means statistically different.

**TABLE 3:** PBSCs *in vitro.*

First author	Species	Number	Character	Type of study	Evaluation method	Injury site(number)	Degree of damage
Frisch, J.(2019)	*in vitro*	NI	4 donors age 42 ± 27	Basic Medical Experiment	Biochemical analyses, Histological and immunohistochemical analyses, Histomorphometry, and Real-time RT-PCR analyses	NI	NA
Hopper, N.(2015)	*in vitro*	NI	12 young (32.9 ± 9.3 years) volunteers	Basic Medical Experiment	Scratch assay, xCELLigence assay, Cell proliferation, Cell proliferation, mRNA expression, PCR array, and Quantitative real-time PCR	NI	NA
Turajane, T.(2014)	*in vitro*	10	10 patients (median age 58 years, range 56–60 years, eight females)	Basic Medical Experiment	Attachment and proliferation assays, Attachment and proliferation assays, Flow cytometry analysis, RT-PCR analysis, Scanning electron microscopy, and Histology	NI	Half ICRS grade = 2 Remainder ICRS grade>3
Kim, J.(2012)	*in vitro*	NI	NA	Basic Medical Experiment	In vitro differentiation, Classification of differentially regulated proteins, western blot, and real-time RT-PCR analysis, and Immunofluorescent-staining	NI	NI
Chong, P. P.(2012)	*in vitro*	NI	NA	Basic Medical Experiment	Biochemical Assays, Morphological Analysis of Chondrogenic, Osteogenic, and Adi-Pyogenic, and Gene Expression Analysis,rt-PCR,	NI	NI
Casado, J. G.(2012)	*in vitro*	NI	Large White pigs aged between 3 and 4 months	Basic Medical Experiment	flow cytometry, adipogenic, chondrogenic and osteogenic differentiation, and Quantitative RT-PCR	NI	NI
Pufe, T.(2008)	*in vitro*	NA	NA	Basic Medical Experiment	Immunohistochemistry, Electron Microscopy, and Enzyme-Linked Immunosorbent Assay,rt-PCR,	NI	NI

First author	Cell source	Cultivation and extraction methods	Cell character markers	Number of cells	Cell implantation method	Surgical approach
Frisch, J.(2019)	rAAV transferred PB MSC	DGC and PA	IGF-I, aggrecan, COL2A1, and SOX9	NA	NA	NA
Hopper, N.(2015)	PBSCs	DGC	C5a, CXCL1, ICAM-1, IL-1β, IL-1ra, IL-6, IL-8, IL-13, IL-16, CXCL10, CXCL11, CCL2, MIF, CCL5, and PAI-1	NA	NA	NA
Turajane, T.(2014)	Autologous G-CSFactivated PB	DGC	*CD34*, *CD29*, *CD44*, *CD45*, *CD90*, *and CD105*	NA	NA	NA
Kim, J.(2012)	PBMSC	NA	*CD34*, *CD45*. *CD133*, *CD73*, *CD90,CD105*,PDGF-B, and HLA-ABC	NA	NI	NI
Chong, P. P.(2012)	PBMSC	NA	*CD105*, *CD166*, *and CD29*	NA	NI	NI
Casado, J. G.(2012)	Peripheral blood mononuclear cells	Erythrocyte lysis, DGC and PA	*CD29* ^*+*^, *CD44* ^*+*^, *CD45* ^*−*^, *CD90* ^*+*^, and *CD105+*	NA	NI	NI
Pufe, T.(2008)	Peripheral blood mononuclear cells	DGC and PA	NA	NA	NA	NA

First author	Time	Postoperative treatment	Clinical outcome	Imaging results	Experimental results	Adverse event
Frisch, J.(2019)	NA	NI	NI	NI	Enhanced proliferative and chondrogenic activities, Significantly increased cellularity, Enhanced levels of chondrogenic marker expression	NA
Hopper, N.(2015)	NA	NI	NI	NI	The wound closure rate at the 3 h time point was significantly higher* no significant difference between the PBMC-stimulated and non-stimulated test groups in the total DNA amount The mRNA levels for these genes were upregulated by the 24 h PBMC stimulus: SOX9 and COL2A1	NA
Turajane, T.(2014)	7 days	NI	NI	NI	Cell proliferation on day 7 showed statistically significant differences* increase in cell attachment and cell proliferation* Sox9 increased in days 7 and 14, Sox9 increased on days 7, Aggrecan increases were statistically significant on days 7 and 14	NA
Kim, J.(2012)	NA	NI	NA	NI	MSC from PB also shows the differentiation of chondroitin	NA
Chong, P. P.(2012)	NA	NI	NA	NI	MSCs from PB maintain similar characteristics and have similar chondrogenic differentiation potential to those derived from BM while producing comparable s-GAG expressions to chondrocytes	NA
Casado, J. G.(2012)	2 weeks	NI	NA	NI	PBMSC have both chondrogenic and adipogenic potential	NA
Pufe, T.(2008)	6 weeks	NI	NI	NI	A strong accumulation of collagen type II after a 6 weeks found chondrogenic differentiation with a continuous expression of collagen type II mRNA and protein	NA

* means statistically different.

**TABLE 4:** QUADAS quality assessment of other study(Y =Yes, N=No, and U=Unclear) based on the items that are described in the method section.

items First author	1	2	3	4	5	6	7	8	9	10	11	12	13	14	15	16	17	18
Henson, F. (2021)	Y	Y	Y	Y	U	N	N	N	Y	Y	Y	Y	Y	Y	U	N	N	N
Ying, J.(2020)	Y	Y	Y	U	Y	Y	Y	Y	Y	Y	Y	Y	Y	N	N	Y	Y	U
Monckeberg, J. E. (2019)	Y	Y	Y	Y	Y	Y	Y	Y	N	N	U	Y	Y	Y	Y	Y	Y	Y
Broeckx, S. Y.(2019)	Y	Y	U	N	N	N	Y	Y	Y	Y	Y	N	N	Y	Y	Y	Y	Y
Broeckx, S. Y.(2019)	Y	N	N	U	Y	Y	Y	Y	Y	Y	Y	Y	N	N	Y	N	N	N
Frisch, J.(2019)	Y	Y	Y	Y	N	Y	Y	Y	Y	Y	N	N	U	Y	U	U	U	U
Hopper, N.(2015)	Y	Y	N	Y	Y	Y	N	Y	N	Y	U	Y	Y	U	Y	Y	U	U
Turajane, T.(2014)	N	N	Y	N	N	Y	Y	N	N	U	Y	Y	Y	U	U	Y	U	N
Fu, W. L.(2014)	Y	Y	N	N	Y	Y	N	Y	Y	N	U	Y	Y	Y	Y	N	Y	U
Fu, W. L.(2014)	Y	Y	Y	Y	Y	U	Y	N	N	U	Y	Y	Y	Y	Y	Y	Y	U
Broeckx, S.(2014)	U	Y	N	N	Y	Y	U	Y	Y	Y	Y	Y	Y	U	Y	Y	Y	N
Broeckx, S.(2014)	Y	Y	N	N	Y	Y	N	Y	Y	Y	Y	Y	Y	Y	Y	Y	Y	Y
Turajane, T.(2013)	Y	Y	Y	Y	N	Y	Y	U	Y	N	Y	Y	Y	U	U	Y	Y	Y
Skowroński, J.(2013)	Y	Y	Y	Y	Y	N	Y	Y	U	U	U	N	Y	Y	U	U	U	U
Saw, K. Y.(2013)	Y	N	Y	U	U	U	Y	Y	U	Y	N	Y	N	N	Y	U	Y	U
Skowroński, J.(2012)	N	N	N	Y	U	Y	Y	U	U	U	U	Y	Y	Y	Y	Y	Y	Y
Kim, J.(2012)	N	Y	N	N	U	Y	Y	Y	Y	Y	Y	Y	Y	N	Y	U	U	U
Chong, P. P.(2012)	U	Y	Y	U	Y	Y	Y	U	Y	N	N	Y	Y	U	Y	Y	N	Y
Casado, J. G.(2012)	U	N	Y	Y	N	Y	N	U	U	Y	Y	Y	Y	U	Y	Y	Y	Y
Saw, K. Y.(2012)	Y	Y	Y	N	U	N	Y	Y	U	Y	Y	Y	N	U	Y	Y	N	Y
Pufe, T.(2008)	Y	Y	Y	Y	N	N	Y	U	U	Y	Y	Y	Y	Y	U	U	N	U
Jancewicz, P.(2004)	Y	Y	N	N	Y	U	Y	Y	Y	Y	Y	Y	Y	N	Y	U	Y	N

### 3.2 PBMSC in Humans

We included nine studies with human subjects, including one prospective study, three comparative studies, 4 case reports, and one RCTs (Jancewicz et al., 1995; Saw et al., 2011; Skowroński et al., 2012; Saw et al., 2013; Skowroński and Rutka, 2013; Fu et al., 2014b; Turajane et al., 2014; Monckeberg et al., 2019; Ying et al., 2020). A total of 225 people were included. Most injuries are concentrated in the patella and femoral condyle, and a few in the hip joint. Cartilage damage in all patients included in the study was degenerative. Except for the study conducted by Ying et al. (2020), which did not report the degree of cartilage damage, the rest of the studies reported that cartilage damage and the ICRS grade was greater than grade 3. The evaluation methods include International Knee Documentation Committee score (IKDC), visual analog scale (VAS), and International Cartilage Repair Society morphologic score system (ICRS) for subjective scoring; X-ray and Magnetic Resonance Imaging (MRI) for imaging examination; and tissue biopsy and Immunohistochemistry for laboratory examination. Seven studies used the G-CSF-stimulated peripheral blood stem cells, and two studies used apheresis peripheral blood stem cells. The preparation process uses red blood cell lysis and gradient centrifugation, which has been proven to be effective in isolating PBMSCs (Kim et al., 2012). All studies used the intra-articular injection for cell implantation. Five articles report on postoperative treatment, including drug therapy: acetaminophen, NSAIDs, and Dexmedetomidine, and different types of rehabilitation programs. Seven studies reported clinical outcomes, except Ying, J (Ying et al., 2020), who reported no significant difference in HHSs between the two groups at 36 months followup. However, clinical results of the remaining six studies reported a significant improvement in clinical scores (KOOS, VAS, The Western Ontario, and McMaster Universities Osteoarthritis Index (WOMAC)) after the peripheral blood-derived stem cells were injected into the defect site. Similarly, in the imaging results and laboratory test results, except for Ying, J, all the other reported studies showed a statistically significant improvement after peripheral blood stem cell transplantation. In terms of adverse events, except for a case of deep vein thrombosis reported by Saw, K. Y, which is a high-risk event, all the other adverse events are low-risk events, including fever and joint adhesion (Saw et al., 2013). The detailed information is shown in Table 2.

### 3.3 PBMSC in Animals

Six animal studies were included in our systematic review, including two RCTs, one comparative study, one controlled laboratory study, one preliminary study, and one pilot study (Broeckx et al., 2014a; Fu et al., 2014a; Broeckx et al., 2014b; Broeckx et al., 2019a; Monckeberg et al., 2019; Henson et al., 2021), and subjects included sheep, horses, and rabbits. The lesions are mainly concentrated in the lower extremity joints or the metacarpophalangeal joints. The cartilage defects of the experimental subjects of Fu, W. L (Fu et al., 2014a), Henson, F. (Henson et al., 2021), and Broeckx, S. Y (Broeckx et al., 2019b) were all using experimental modeling, and the cartilage defects of the experimental subjects of other researchers were all caused by degenerative diseases. Grade of cartilage damage was not reported. Two studies used the G-CSF-stimulated peripheral blood stem cells, and four studies used chondrogenic induced PBMSCs. Gradient centrifugation was used for cell isolation in all experiments, and plastic adhesion was also used in some experiments. All studies did not impose strict requirements on the postoperative rehabilitation of experimental animals and did not limit their range and intensity of activities. Only Broeckx, S. Y. in the 2019 experiment gave experimental animals postoperative drug treatment for sedation and analgesia. In experiments where flow cytometry was performed, Henson, F. et al. (2021) detected: *CD34, CD45, CD73, CD90*,and *CD 105*, and Broeckx, S. Y. et al. (2019) detected: *CD45, MHC II, CD29*, *CD44,* and *CD90*, Fu, W. L. et al. (2014) detected: *CD44, CD45, and MHC II*. All studies used intra-articular injection for cell implantation. In the postoperative results, the clinical results showed a consistent trend of improvement, whether it was objective indicators or subjective scores. In the imaging results, except for the study done by Broeckx, S. Y in 2019, the radiographic changes were not significantly different. The rest showed improvements after the use of peripheral blood-derived stem cells. In the absence of laboratory validation, only three articles showed increased levels of cartilage-related matrix or components around damaged cartilage tissue, such as type II collagen and cartilage oligomeric matrix protein. The detailed information is shown in Table 1.

### 3.4 PBMSC *In Vitro*

In this systematic review, we included a total of seven articles from *in vitro* studies (Pufe et al., 2008; Casado et al., 2012; Chong et al., 2012; Kim et al., 2012; Turajane et al., 2014; Hopper et al., 2015b; Frisch et al., 2017b). Among them, the donors of three experiments were humans, the donors of one experiment were pigs, and the peripheral blood donors were not specified in the remaining experiments. The validation methods for *in vitro* experiments include scratch experiments, immunohistochemistry, flow cytometry, RT-PCR, and more. The authors described the peripheral blood-derived stem cells used in the article, including the peripheral blood mononuclear cells, PBMSC, autologous G-CSF activated PB, and peripheral blood stem cells (PBSCs). CD105+ was found in all experiments with flow cytometry results, but CD34^+^ was found in all experiments by Turajane, T. et al., may indicate that the cells used in the experiments are the nonmesenchymal presence of stem cells. Other experiments uncovered the secretion of many chemokines, which may also be largely involved in the induction of cartilage repair. In terms of results, all studies have proved that the peripheral blood-derived stem cells can differentiate into cartilage and have the potential to repair cartilage damage. Hopper, N, and Turajane, T. all found the upregulation of SOX-9 in their experiments, indicating that the peripheral blood-derived stem cells have a regulatory effect on cartilage differentiation. The formation of the extrachondral matrix was found in all *in vitro* studies, which is important for cartilage repair. The detailed information is shown in Table 3.

## 4 Discussion

According to the research on stem cells derived from peripheral blood *in vitro*, they have the same or similar chondrogenic differentiation ability as that of the bone marrow mesenchymal stem cells in the process of culture and passage *in vitro*, as Chong, P. P. showed in his research. (Chong et al., 2012; Gong et al., 2021). Combined with the human and animal research reports on its improved *in vivo* results, this systematic review shows that peripheral blood-derived stem cells have chondrogenic differentiation ability and can induce chondrogenic differentiation and repair *in vivo*, and have statistical significance in the clinical and imaging prognosis. There is improvement of academic differences. Compared with bone marrow, the peripheral blood is easier to obtain, widely sourced, and simple to obtain. In the future, peripheral blood will be a more potential cell source for cell therapy in the treatment of cartilage damage.

**FIGURE 5:**
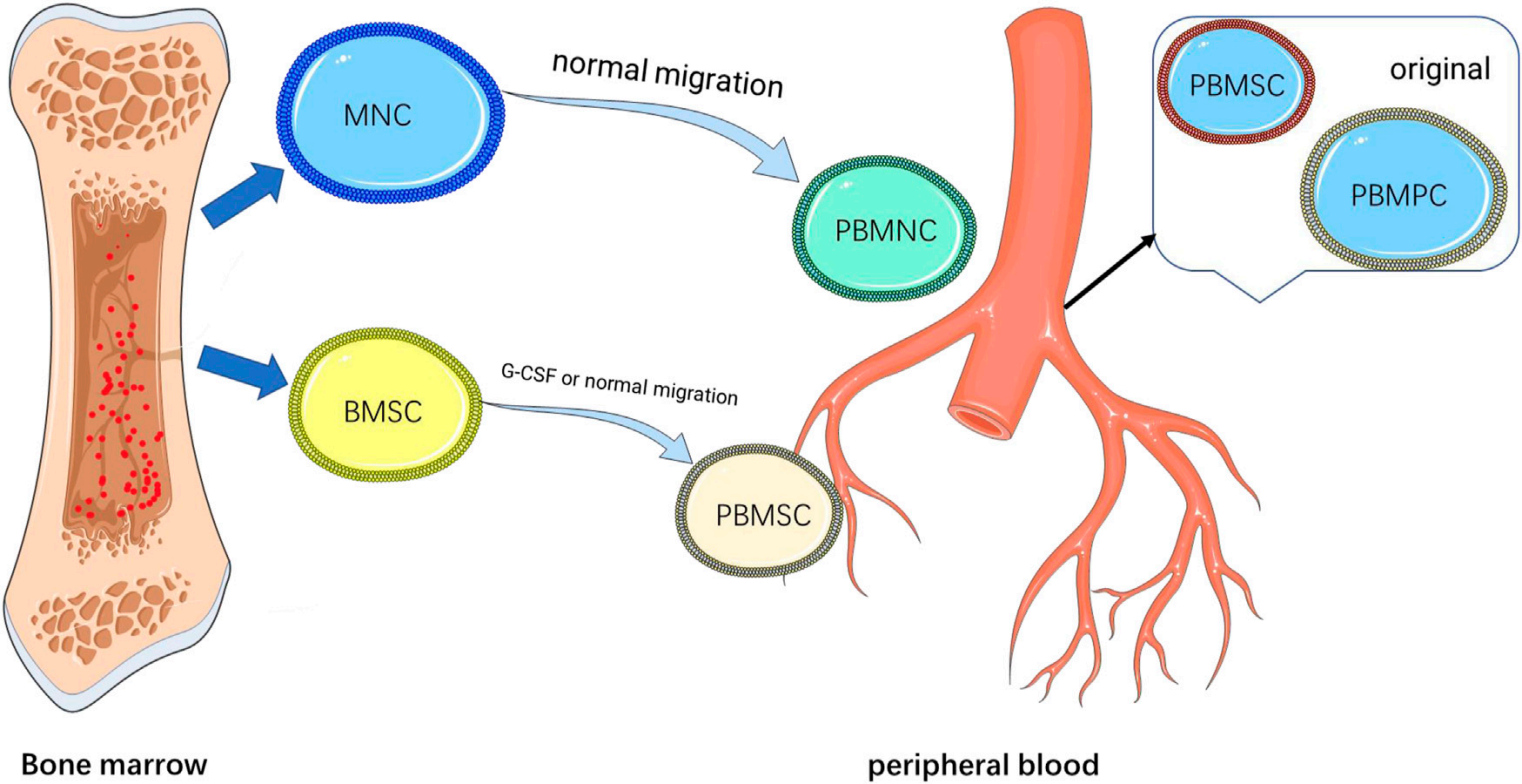
Basic biology of blood-derived stem cells. MNC, mononuclear cells; BMSC, Bone marrow mesenchymal stem cells; PBMSC, peripheral blood mesenchymal stem cells; PBMNC, peripheral blood mononuclear cells; and PBMPC, peripheral blood mesenchymal progenitor cells.

However, some studies have contrary results. In the study of Ying, J. et al. (Ying et al., 2020), peripheral blood-derived stem cells did not show improvement in the clinical and imaging results in the treatment of femoral head necrosis, and combined treatment in histology. The bone destruction in the group was more severe than that in the control group. But a previous study showed that combination therapy with an intra-arterial infusion of PBSCs showed improved the outcomes in patients with early and mid-stage necrosis of the femoral head (Schmitt-Sody et al., 2008; Mao et al., 2015). Considering the advantages of PBSC in easily harvesting and stimulating neovascularization and osteogenesis in the damaged skeletal tissue, PBSC transplantation is a selective approach for the treatment of ONFH (Zhang et al., 2016). In this study, it was used to treat patients with femoral head necrosis with cartilage cap separation, which has exceeded the early and middle stages and is an advanced stage disease (Xiong et al., 2016). At this stage, the active expression of osteoclasts and the widespread occurrence of inflammatory responses lead to irreversible necrosis of the femoral head, which may require more complex mechanisms to explain (Feng et al., 2010). Femoral head necrosis is a complex pathophysiological process involving cartilage, subchondral bone, bone, and surrounding tissues. The repair mechanism of cartilage damaged by the peripheral blood stem cells alone may not be able to offset the overall damage caused by the inflammatory response. Moreover, in this study conducted by Ying, J. et al., although the injection of PBSCs into the internal circumflex artery did not improve the survival rate of femoral head necrosis, it had a good effect on relieving pain and improving the joint function. This result can also reflect that peripheral blood-derived stem cells have a repairing effect on intra-articular cartilage damage, although it cannot be reflected in the histology of this study (Hopson and Siverhus, 1988). This makes us think that in the treatment of some diseases with more complex mechanisms than simple cartilage damage, the use of stem cells derived from peripheral blood alone may not have a good prognosis, and more combined treatment or surgical treatment is needed. But not being able to cure the disease is not the same as denying its effect on the repair of cartilage damage Figure 5. 

The cell types and potential repair mechanisms are detailed in Figure 5, 6. At present, the cell source used in most research is G-CSF activated PB or chondrogenic-induced PBMSCs. It has been demonstrated in the previous literature that G-CSF and CXCR4 antagonists can mobilize mesenchymal stem cells into peripheral blood (Pelus, 2008; Kolonin and Simmons, 2009). It can improve the success rate of subsequent mesenchymal stem cell culture, and the density of mesenchymal stem cells is also an important feature to evaluate cartilage repair. Moreover, in the other literature, a simple injection of G-CSF can make bone marrow and peripheral blood mesenchymal stem cells home to the joint cavity and help cartilage regeneration (Sasaki et al., 2017; Turajane et al., 2017). The literature included in this systematic review also showed that G-CSF activated PB has the potential for chondrogenic differentiation and repair and is a good alternative resource. While chondrogenic-induced PBMSCs secrete more extrachondral matrix including aggrecan, type II collagen, and cartilage oligomeric matrix protein when cultured *in vitro*, which reflects better proliferation ability (Broeckx et al., 2014a) and has been shown in one study to better adhere to cartilage in explant cultures (Spaas et al., 2015). TGF-β, one of the cartilage-stimulating growth factors used in the current study for predifferentiation of chondrocyte differentiation, can reduce the expression of MHC (Berglund et al., 2017). This can reduce the occurrence of inflammatory reactions and reduce the chance of immune rejection (Schnabel et al., 2014). The two preparation methods have their advantages, but there is no research to compare the advantages and disadvantages of the two methods to give guiding opinions. Future research can combine the advantages of the two methods, and it is believed that a more effective new preparation method can be obtained.

**FIGURE 6:**
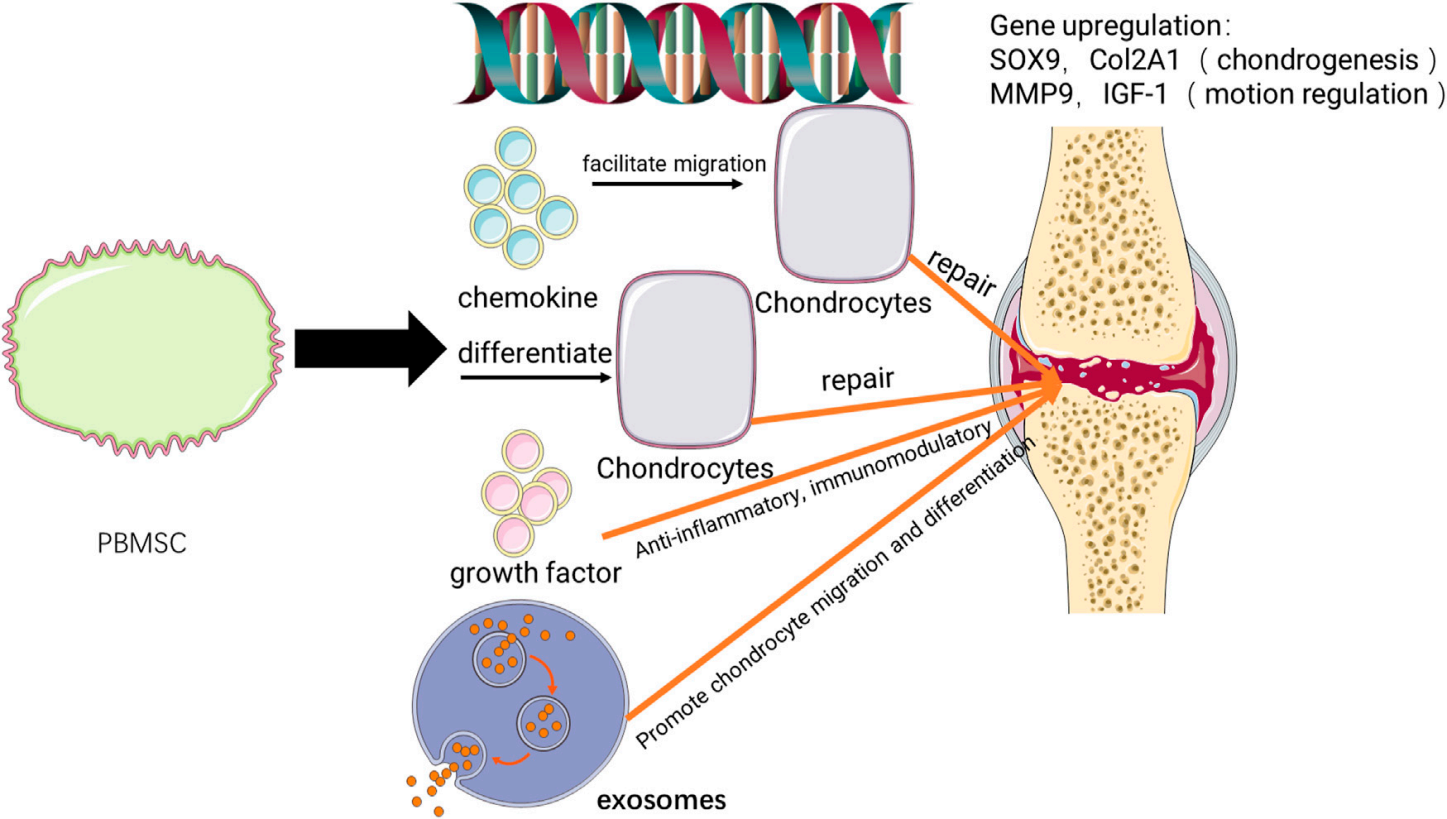
Potential mechanisms of PBMSC in cartilage repair.

Stem cells have many advantages and can effectively treat cartilage damage; for example, they have strong self-renewal capacity, pluripotency, and plasticity. However, the properties of MSCs may be altered by various elements of the local microenvironment that influence differentiation, may cause reduced chondrogenic activity or differentiation into other tissues, so they may suffer from disadvantages such as eventual hypertrophy or tumorigenesis (Chen and Tuan, 2008; Vinatier et al., 2009; Koh et al., 2014; Pandey et al., 2022). However, in the studies we included, adverse events were mild and there was no worsening change in the imaging findings. This may indicate that stem cells derived from peripheral blood have stable differentiation (Chong et al., 2012). This also proves our point that peripheral blood-derived stem cells are an important source of cells to repair cartilage damage.

This article also has certain limitations. In the selection of literature, due to the continuous updating of preparation methods and repair mechanisms, we only included relevant literature after 2008, excluding some studies in older periods, which may make the research results subject to influence. In the statistics of cell phenotype, no further analysis was performed for the events whose cells highly expressed *CD34* ^*+*^ and some studies did not express the mesenchymal stem cell marker *CD105+*. This means that, in some of the included studies, it is not only mesenchymal stem cells that perform cartilage repair, but may also be mononuclear cells or other stem cells in peripheral blood. Therefore, here, we refer to them as the peripheral blood-derived stem cells and use this fully as a resource for cartilage repair.

## 5 Conclusion

Stem cells derived from peripheral blood have the ability to repair cartilage and are an important resource for the treatment of cartilage damage in the future. The specific mechanism and way of repairing cartilage need further study.

## Data Availability

The original contributions presented in the study are included in the article/Supplementary Material. Further inquiries can be directed to the corresponding author.

## References

[B1] Al Faqeh H. Nor Hamdan B. M. Y. Chen H. C. Aminuddin B. S. Ruszymah B. H. I. (2012). The Potential of Intra-articular Injection of Chondrogenic-Induced Bone Marrow Stem Cells to Retard the Progression of Osteoarthritis in a Sheep Model. Exp. Gerontol. 47 (6), 458–464. 10.1016/j.exger.2012.03.018 22759409

[B2] Berglund A. K. Fisher M. B. Cameron K. A. Poole E. J. Schnabel L. V. (2017). Transforming Growth Factor-Β2 Downregulates Major Histocompatibility Complex (MHC) I and MHC II Surface Expression on Equine Bone Marrow-Derived Mesenchymal Stem Cells without Altering Other Phenotypic Cell Surface Markers. Front. Vet. Sci. 4, 84. 10.3389/fvets.2017.00084 28660198 PMC5466990

[B3] Bieback K. Kern S. Kocaömer A. Ferlik K. Bugert P. (2008). Comparing Mesenchymal Stromal Cells from Different Human Tissues: Bone Marrow, Adipose Tissue and Umbilical Cord Blood. Biomed. Mater Eng. 18 (1 Suppl. l), S71–S76. 18334717

[B4] Brittberg M. Lindahl A. Nilsson A. Ohlsson C. Isaksson O. Peterson L. (1994). Treatment of Deep Cartilage Defects in the Knee with Autologous Chondrocyte Transplantation. N. Engl. J. Med. 331 (14), 889–895. 10.1056/nejm199410063311401 8078550

[B5] Broeckx S. Suls M. Beerts C. Vandenberghe A. Seys B. Wuertz-Kozak K. (2014). Allogenic Mesenchymal Stem Cells as a Treatment for Equine Degenerative Joint Disease: a Pilot Study. Cscr 9 (6), 497–503. 10.2174/1574888x09666140826110601 25175766

[B6] Broeckx S. Y. Martens A. M. Bertone A. L. Van Brantegem L. Duchateau L. Van Hecke L. (2019). The Use of Equine Chondrogenic‐induced Mesenchymal Stem Cells as a Treatment for Osteoarthritis: A Randomised, Double‐blinded, Placebo‐controlled Proof‐of‐concept Study. Equine Vet. J. 51 (6), 787–794. 10.1111/evj.13089 30815897 PMC6850029

[B7] Broeckx S. Y. Seys B. Suls M. Vandenberghe A. Mariën T. Adriaensen E. (2019). Equine Allogeneic Chondrogenic Induced Mesenchymal Stem Cells Are an Effective Treatment for Degenerative Joint Disease in Horses. Stem cells Dev. 28 (6), 410–422. 10.1089/scd.2018.0061 30623737 PMC6441287

[B8] Broeckx S. Zimmerman M. Crocetti S. Suls M. Mariën T. Ferguson S. J. (2014). Regenerative Therapies for Equine Degenerative Joint Disease: a Preliminary Study. PloS one 9 (1), e85917. 10.1371/journal.pone.0085917 24465787 PMC3896436

[B9] Casado J. G. Gomez-Mauricio G. Alvarez V. Mijares J. Tarazona R. Bernad A. (2012). Comparative Phenotypic and Molecular Characterization of Porcine Mesenchymal Stem Cells from Different Sources for Translational Studies in a Large Animal Model. Vet. Immunol. Immunopathol. 147 (1-2), 104–112. 10.1016/j.vetimm.2012.03.015 22521281

[B10] Chen F. H. Tuan R. S. (2008). Mesenchymal Stem Cells in Arthritic Diseases. Arthritis Res. Ther. 10 (5), 223. 10.1186/ar2514 18947375 PMC2592798

[B11] Chong P.-P. Selvaratnam L. Abbas A. A. Kamarul T. (2012). Human Peripheral Blood Derived Mesenchymal Stem Cells Demonstrate Similar Characteristics and Chondrogenic Differentiation Potential to Bone Marrow Derived Mesenchymal Stem Cells. J. Orthop. Res. 30 (4), 634–642. 10.1002/jor.21556 21922534

[B12] Emadedin M. Aghdami N. Taghiyar L. Fazeli R. Moghadasali R. Jahangir S. (2012). Intra-articular Injection of Autologous Mesenchymal Stem Cells in Six Patients with Knee Osteoarthritis. Arch. Iran. Med. 15 (7), 422–428. 10.12157/AIM.0010 22724879

[B13] Feng Y. Yang S.-H. Xiao B.-J. Xu W.-H. Ye S.-N. Xia T. (2010). Decreased in the Number and Function of Circulation Endothelial Progenitor Cells in Patients with Avascular Necrosis of the Femoral Head. Bone 46 (1), 32–40. 10.1016/j.bone.2009.09.001 19747991

[B14] Ferris D. J. Frisbie D. D. McIlwraith C. W. Kawcak C. E. (2011). Current Joint Therapy Usage in Equine Practice: a Survey of Veterinarians 2009. Equine veterinary J. 43 (5), 530–535. 10.1111/j.2042-3306.2010.00324.x 21668486

[B15] Frisbie D. D. Kisiday J. D. Kawcak C. E. Werpy N. M. McIlwraith C. W. (2009). Evaluation of Adipose-Derived Stromal Vascular Fraction or Bone Marrow-Derived Mesenchymal Stem Cells for Treatment of Osteoarthritis. J. Orthop. Res. 27 (12), 1675–1680. 10.1002/jor.20933 19544397

[B16] Frisch J. Orth P. Rey-Rico A. Venkatesan J. K. Schmitt G. Madry H. (2017). Peripheral Blood Aspirates Overexpressing IGF-IviarAAV Gene Transfer Undergo Enhanced Chondrogenic Differentiation Processes. J. Cell. Mol. Med. 21 (11), 2748–2758. 10.1111/jcmm.13190 28467017 PMC5661259

[B17] Frisch J. Orth P. Venkatesan J. K. Rey-Rico A. Schmitt G. Kohn D. (2017). Genetic Modification of Human Peripheral Blood Aspirates Using Recombinant Adeno-Associated Viral Vectors for Articular Cartilage Repair with a Focus on Chondrogenic Transforming Growth Factor-β Gene Delivery. Stem cells Transl. Med. 6 (1), 249–260. 10.5966/sctm.2016-0149 28170175 PMC5442727

[B18] Fu W.-L. Ao Y.-F. Ke X.-Y. Zheng Z.-Z. Gong X. Jiang D. (2014). Repair of Large Full-Thickness Cartilage Defect by Activating Endogenous Peripheral Blood Stem Cells and Autologous Periosteum Flap Transplantation Combined with Patellofemoral Realignment. Knee 21 (2), 609–612. 10.1016/j.knee.2013.10.010 24405791

[B19] Fu W.-L. Zhou C.-Y. Yu J.-K. (2014). A New Source of Mesenchymal Stem Cells for Articular Cartilage Repair. Am. J. Sports Med. 42 (3), 592–601. 10.1177/0363546513512778 24327479

[B20] Gong J. Fairley J. Cicuttini F. M. Hussain S. M. Vashishtha R. Chou L. (2021). Effect of Stem Cell Injections on Osteoarthritis-Related Structural Outcomes: A Systematic Review. J. Rheumatol. 48 (4), 585–597. 10.3899/jrheum.200021 33004537

[B21] Henson F. Lydon H. Birch M. Brooks R. McCaskie A. (2021). Using Apheresis‐derived Cells to Augment Microdrilling in the Treatment of Chondral Defects in an Ovine Model. J. Orthop. Res. 39 (7), 1411–1422. 10.1002/jor.24889 33146412 PMC7612025

[B22] Hopper N. Henson F. Brooks R. Ali E. Rushton N. Wardale J. (2015). Peripheral Blood Derived Mononuclear Cells Enhance Osteoarthritic Human Chondrocyte Migration. Arthritis Res. Ther. 17 (1), 199. 10.1186/s13075-015-0709-z 26249339 PMC4528856

[B23] Hopper N. Wardale J. Brooks R. Power J. Rushton N. Henson F. (2015). Peripheral Blood Mononuclear Cells Enhance Cartilage Repair in *In Vivo* Osteochondral Defect Model. PloS one 10 (8), e0133937. 10.1371/journal.pone.0133937 26252391 PMC4529143

[B24] Hopson C. N. Siverhus S. W. (1988). Ischemic Necrosis of the Femoral Head. Treatment by Core Decompression. J. Bone & Jt. Surg. 70 (7), 1048–1051. 10.2106/00004623-198870070-00013 3403573

[B25] Huang Y.-C. Yang Z.-M. Chen X.-H. Tan M.-Y. Wang J. Li X.-Q. (2009). Isolation of Mesenchymal Stem Cells from Human Placental Decidua Basalis and Resistance to Hypoxia and Serum Deprivation. Stem Cell. Rev Rep 5 (3), 247–255. 10.1007/s12015-009-9069-x 19590988

[B26] Hunziker E. B. (2002). Articular Cartilage Repair: Basic Science and Clinical Progress. A Review of the Current Status and Prospects. Osteoarthr. Cartil. 10 (6), 432–463. 10.1053/joca.2002.0801 12056848

[B27] Jancewicz P. Dzienis W. Pietruczuk M. Skowroński J. Bielecki M. (1995). Osteochondral Defects of the Talus Treated by Mesenchymal Stem Cell Implantation-Eearly Results. Rocz. Akad. Med. Bialymst 49 (Suppl. 1), 25–27. 15638364

[B28] Jiang Y. Tuan R. S. (2015). Origin and Function of Cartilage Stem/progenitor Cells in Osteoarthritis. Nat. Rev. Rheumatol. 11 (4), 206–212. 10.1038/nrrheum.2014.200 25536487 PMC5413931

[B29] Kassis I. Zangi L. Rivkin R. Levdansky L. Samuel S. Marx G. (2006). Isolation of Mesenchymal Stem Cells from G-CSF-Mobilized Human Peripheral Blood Using Fibrin Microbeads. Bone Marrow Transpl. 37 (10), 967–976. 10.1038/sj.bmt.1705358 16670702

[B30] Kim J. Shin J. M. Jeon Y. J. Chung H. M. Chae J.-I. (2012). Proteomic Validation of Multifunctional Molecules in Mesenchymal Stem Cells Derived from Human Bone Marrow, Umbilical Cord Blood and Peripheral Blood. PloS one 7 (5), e32350. 10.1371/journal.pone.0032350 22615730 PMC3353928

[B31] Koh Y. G. Choi Y. J. Kwon O. R. Kim Y. S. (2014). Second-Look Arthroscopic Evaluation of Cartilage Lesions after Mesenchymal Stem Cell Implantation in Osteoarthritic Knees. Am. J. Sports Med. 42 (7), 1628–1637. 10.1177/0363546514529641 24743139

[B32] Kolonin M. G. Simmons P. J. (2009). Combinatorial Stem Cell Mobilization. Nat. Biotechnol. 27 (3), 252–253. 10.1038/nbt0309-252 19270674

[B33] Larochelle A. Krouse A. Metzger M. Orlic D. Donahue R. E. Fricker S. (2006). AMD3100 Mobilizes Hematopoietic Stem Cells with Long-Term Repopulating Capacity in Nonhuman Primates. Blood 107 (9), 3772–3778. 10.1182/blood-2005-09-3592 16439684 PMC1895780

[B34] Madry H. Grün U. W. Knutsen G. (2011). Cartilage Repair and Joint Preservation. Dtsch. Arzteblatt Int. 108 (40), 669–677. 10.3238/arztebl.2011.0669 PMC3221423 22114626

[B35] Mao Q. Wang W. Xu T. Zhang S. Xiao L. Chen D. (2015). Combination Treatment of Biomechanical Support and Targeted Intra-arterial Infusion of Peripheral Blood Stem Cells Mobilized by Granulocyte-Colony Stimulating Factor for the Osteonecrosis of the Femoral Head: a Randomized Controlled Clinical Trial. J. bone mineral Res. official J. Am. Soc. Bone Mineral Res. 30 (4), 647–656. 10.1002/jbmr.2390 PMC4376653 25349059

[B36] Moher D. Liberati A. Tetzlaff J. Altman D. G. (2009). Preferred Reporting Items for Systematic Reviews and Meta-Analyses: the PRISMA Statement. PLoS Med. 6 (7), e1000097. 10.1371/journal.pmed.1000097 19621072 PMC2707599

[B37] Monckeberg J. E. Rafols C. Apablaza F. Gerhard P. Rosales J. (2019). Intra-articular Administration of Peripheral Blood Stem Cells with Platelet-Rich Plasma Regenerated Articular Cartilage and Improved Clinical Outcomes for Knee Chondral Lesions. Knee 26 (4), 824–831. 10.1016/j.knee.2019.05.008 31227435

[B38] Orth P. Rey-Rico A. Venkatesan J. K. Madry H. Cucchiarini M. (2014). Current Perspectives in Stem Cell Research for Knee Cartilage Repair. Stem Cells Cloning 7, 1–17. 10.2147/SCCAA.S42880 24520197 PMC3897321

[B39] Pandey V. Madi S. Gupta P. (2022). The Promising Role of Autologous and Allogeneic Mesenchymal Stromal Cells in Managing Knee Osteoarthritis. What Is beyond Mesenchymal Stromal Cells? J. Clin. Orthop. trauma 26, 101804. 10.1016/j.jcot.2022.101804 35242531 PMC8857498

[B40] Pelus L. M. (2008). Peripheral Blood Stem Cell Mobilization: New Regimens, New Cells, where Do We Stand. Curr. Opin. Hematol. 15 (4), 285–292. 10.1097/moh.0b013e328302f43a 18536564 PMC2806229

[B41] Pufe T. Petersen W. Fändrich F. Varoga D. Wruck C. J. Mentlein R. (2008). Programmable Cells of Monocytic Origin (PCMO): a Source of Peripheral Blood Stem Cells that Generate Collagen Type II-Producing Chondrocytes. J. Orthop. Res. 26 (3), 304–313. 10.1002/jor.20516 17963214

[B42] Rackwitz L. Djouad F. Janjanin S. Nöth U. Tuan R. S. (2014). Functional Cartilage Repair Capacity of De-differentiated, Chondrocyte- and Mesenchymal Stem Cell-Laden Hydrogels *In Vitro* . Osteoarthr. Cartil. 22 (8), 1148–1157. 10.1016/j.joca.2014.05.019 PMC5398282 24887551

[B43] Raghunath J. Sutherland J. Salih V. Mordan N. Butler P. E. Seifalian A. M. (2010). Chondrogenic Potential of Blood-Acquired Mesenchymal Progenitor Cells. J. Plastic, Reconstr. Aesthetic Surg. 63 (5), 841–847. 10.1016/j.bjps.2009.01.063 19345657

[B44] Reissis D. Tang Q. O. Cooper N. C. Carasco C. F. Gamie Z. Mantalaris A. (2016). Current Clinical Evidence for the Use of Mesenchymal Stem Cells in Articular Cartilage Repair. Expert Opin. Biol. Ther. 16 (4), 535–557. 10.1517/14712598.2016.1145651 26798997

[B45] Saris D. B. Vanlauwe J. Victor J. Almqvist K. F. Verdonk R. Bellemans J. (2009). Treatment of Symptomatic Cartilage Defects of the Knee: Characterized Chondrocyte Implantation Results in Better Clinical Outcome at 36 Months in a Randomized Trial Compared to Microfracture. Am. J. Sports Med. 37 Suppl 1 (Suppl. 1), 10s–19s. 10.1177/0363546509350694 19846694

[B46] Sasaki T. Akagi R. Akatsu Y. Fukawa T. Hoshi H. Yamamoto Y. (2017). The Effect of Systemic Administration of G-CSF on a Full-Thickness Cartilage Defect in a Rabbit Model MSC Proliferation as Presumed Mechanism. Bone & Jt. Res. 6 (3), 123–131. 10.1302/2046-3758.63.bjr-2016-0083 PMC5376658 28258115

[B47] Saw K.-Y. Anz A. Merican S. Tay Y.-G. Ragavanaidu K. Jee C. S. Y. (2011). Articular Cartilage Regeneration with Autologous Peripheral Blood Progenitor Cells and Hyaluronic Acid after Arthroscopic Subchondral Drilling: a Report of 5 Cases with Histology. Arthrosc. J. Arthrosc. Relat. Surg. 27 (4), 493–506. 10.1016/j.arthro.2010.11.054 21334844

[B48] Saw K.-Y. Anz A. Siew-Yoke Jee C. Merican S. Ching-Soong Ng R. Roohi S. A. (2013). Articular Cartilage Regeneration with Autologous Peripheral Blood Stem Cells versus Hyaluronic Acid: a Randomized Controlled Trial. Arthrosc. J. Arthrosc. Relat. Surg. 29 (4), 684–694. 10.1016/j.arthro.2012.12.008 23380230

[B49] Schmitt-Sody M. Kirchhoff C. Mayer W. Goebel M. Jansson V. (2008). Avascular Necrosis of the Femoral Head: Inter- and Intraobserver Variations of Ficat and ARCO Classifications. Int. Orthop. 32 (3), 283–287. 17396260 10.1007/s00264-007-0320-2 PMC2323428

[B50] Schnabel L. V. Pezzanite L. M. Antczak D. F. Felippe M. J. B. Fortier L. A. (2014). Equine Bone Marrow-Derived Mesenchymal Stromal Cells Are Heterogeneous in MHC Class II Expression and Capable of Inciting an Immune Response *In Vitro* . Stem Cell. Res. Ther. 5 (1), 13. 10.1186/scrt402 24461709 PMC4055004

[B51] Seol D. McCabe D. J. Choe H. Zheng H. Yu Y. Jang K. (2012). Chondrogenic Progenitor Cells Respond to Cartilage Injury. Arthritis & Rheumatism 64 (11), 3626–3637. 10.1002/art.34613 22777600 PMC4950521

[B52] Skowroński J. Rutka M. (2013). Osteochondral Lesions of the Knee Reconstructed with Mesenchymal Stem Cells - Results. Ortop. Traumatol. Rehabil. 15 (3), 195–204. 10.5604/15093492.1058409 23897996

[B53] Skowroński J. Skowroński R. Rutka M. (2012). Cartilage Lesions of the Knee Treated with Blood Mesenchymal Stem Cells - Results. Ortop. Traumatol. Rehabil. 14 (6), 569–577. 10.5604/15093492.1012404 23382284

[B54] Spaas J. H. Broeckx S. Y. Chiers K. Ferguson S. J. Casarosa M. Van Bruaene N. (2015). Chondrogenic Priming at Reduced Cell Density Enhances Cartilage Adhesion of Equine Allogeneic MSCs - a Loading Sensitive Phenomenon in an Organ Culture Study with 180 Explants. Cell. Physiol. Biochem. 37 (2), 651–665. 10.1159/000430384 26344791

[B55] Turajane T. Chaveewanakorn U. Fongsarun W. Aojanepong J. Papadopoulos K. I. (2017). Avoidance of Total Knee Arthroplasty in Early Osteoarthritis of the Knee with Intra-articular Implantation of Autologous Activated Peripheral Blood Stem Cells versus Hyaluronic Acid: A Randomized Controlled Trial with Differential Effects of Growth Factor Addition. Stem Cells Int. 2017, 8925132. 10.1155/2017/8925132 29056974 PMC5625803

[B56] Turajane T. Chaweewannakorn U. Larbpaiboonpong V. Aojanepong J. Thitiset T. Honsawek S. (2013). Combination of Intra-articular Autologous Activated Peripheral Blood Stem Cells with Growth Factor Addition/ Preservation and Hyaluronic Acid in Conjunction with Arthroscopic Microdrilling Mesenchymal Cell Stimulation Improves Quality of Life and Regenerates Articular Cartilage in Early Osteoarthritic Knee Disease. J. Med. Assoc. Thai 96 (5), 580–588. 23745314

[B57] Turajane T. Thitiset T. Honsawek S. Chaveewanakorn U. Aojanepong J. Papadopoulos K. I. (2014). Assessment of Chondrogenic Differentiation Potential of Autologous Activated Peripheral Blood Stem Cells on Human Early Osteoarthritic Cancellous Tibial Bone Scaffold. Musculoskelet. Surg. 98 (1), 35–43. 10.1007/s12306-013-0303-y 24178764

[B58] Vinatier C. Bouffi C. Merceron C. Gordeladze J. Brondello J.-M. Jorgensen C. (2009). Cartilage Tissue Engineering: towards a Biomaterial-Assisted Mesenchymal Stem Cell Therapy. Cscr 4 (4), 318–329. 10.2174/157488809789649205 PMC3218613 19804369

[B59] Xiong M. Y. Liu L. Q. Liu S. Q. Liu Z. H. Gao H. F. (2016). Effects of Osteoprotegerin, RANK and RANKL on Bone Destruction and Collapse in Avascular Necrosis Femoral Head. Am. J. Transl. Res. 8 (7), 3133–3140. 27508034 PMC4969450

[B60] Ying J. Wang P. Ding Q. Shen J. O'Keefe R. J. Chen D. (2020). Peripheral Blood Stem Cell Therapy Does Not Improve Outcomes of Femoral Head Osteonecrosis with Cap‐Shaped Separated Cartilage Defect. J. Orthop. Res. 38 (2), 269–276. 10.1002/jor.24471 31520480

[B61] Zhang Y. Yin J. Ding H. Zhang C. Gao Y.-S. (2016). Vitamin K2 Ameliorates Damage of Blood Vessels by Glucocorticoid: a Potential Mechanism for its Protective Effects in Glucocorticoid-Induced Osteonecrosis of the Femoral Head in a Rat Model. Int. J. Biol. Sci. 12 (7), 776–785. 10.7150/ijbs.15248 27313492 PMC4910597

[B62] Zvaifler N. J. Marinova-Mutafchieva L. Adams G. Edwards C. J. Moss J. Burger J. A. (2000). Mesenchymal Precursor Cells in the Blood of Normal Individuals. Arthritis Res. Ther. 2 (6), 477–488. 10.1186/ar130 PMC17820 11056678

